# Potential of Assistive Robots in Clinical Nursing: An Observational Study of Nurses’ Transportation Tasks in Rural Clinics of Bavaria, Germany

**DOI:** 10.3390/nursrep14010021

**Published:** 2024-01-24

**Authors:** Domenic Sommer, Jakob Kasbauer, Dietmar Jakob, Sebastian Schmidt, Florian Wahl

**Affiliations:** Technology Campus Grafenau, Deggendorf Institute of Technology, 94481 Grafenau, Germany; jakob.kasbauer@th-deg.de (J.K.); dietmar.jakob@th-deg.de (D.J.); sebastian.schmidt@th-deg.de (S.S.); florian.wahl@th-deg.de (F.W.)

**Keywords:** logistics, nursing task analysis, intra-hospital transfer, time and motion study, assistive

## Abstract

Transportation tasks in nursing are common, often overlooked, and directly impact patient care time in the context of staff shortages and an aging society. Current studies lack a specific focus on transportation tasks, a gap our research aims to fill. By providing detailed data on transportation needs in nursing, our study establishes a crucial foundation for the development and integration of assistive robots in clinical settings. In July and September 2023, we conducted weekly observations of nurses to assess clinical transportation needs. We aim to understand the economic impact and the methods nurses use for transportation tasks. We conducted a participant observation using a standardized app-based form over a seven-day observation period in two rural clinics. N = 1830 transports were made by nurses and examined by descriptive analysis. Non-medical supplies account for 27.05% (n = 495) of all transports, followed by medical supplies at 17.32% (n = 317), pharmacotherapy at 14.10% (n = 258) and other other categories like meals or drinks contributing 12.68% (n = 232). Most transports had a factual transport time of under a minute, with patient transport and lab samples displaying more variability. In total, 77.15% of all transports were made by hand. Requirements to collect items or connect transports with patient care were included in 5% of all transports. Our economic evaluation highlighted meals as the most costly transport, with 9596.16 € per year in the observed clinics. Budget-friendly robots would amortize these costs over one year by transporting meals. We support understanding nurses’ transportation needs via further research on assistive robots to validate our findings and determine the feasibility of transport robots.

## 1. Introduction

Germany, like many EU nations, faces healthcare challenges due to an aging population and increasing multi-morbidity [1,2]. As of 2023, approximately 20% of the German population is over the age of 65, a figure projected to increase by 2050 [3,4]. The COVID-19 pandemic has further escalated the demand for health services, simultaneously highlighting the acute shortage of nursing staff [5]. The nursing sector has started to struggle to fulfill the escalating care demands driven by demographic transformations, particularly in rural areas [6,7]. Additionally, the challenges will extend, as projections indicate that by 2023, Germany could face a shortfall of approximately 500,000 nurses [6,8]. This challenge is compounded by the need to manage rising health expenditures, prompting governmental plans to curtail funding for support staff [9,10]. Human support workers, in the form of transport and delivery services or care assistants, have a vital role but also face staff shortages and are limited due to additional costs for nursing wards [11].

Current healthcare challenges regarding nurses’ health can be extended, which are demanding and lead to intentions to leave the profession [12]. Transportation tasks, such as the transfer of patients and equipment, are commonplace in clinical settings [13,14,15]. Nurses transfer patients more than 40 times during their shift, significantly contributing to nurses’ physical strain [14]. As transportation is repetitive and includes heavy loads, musculoskeletal diseases and back pain are prevalent issues among nurses [14].

The urgency to implement efficient technological solutions that relieve nurses’ burden, save costs, and augment capacities regarding the mentioned challenges becomes paramount [16,17]. Transportation tasks are widespread in nursing, reducing the time for direct patient care, and do not always need human execution. Among technical solutions, assistive robots promise to alleviate, support, and free-up capacity in repetitive clinic processes [17,18,19,20]. Robots can take over routine transportation tasks, such as lab specimens and patient forms, which ties up nursing staff and detracts from time devoted to patient care, treatment, and therapy [21,22].

Understanding the current state of nurse transportation tasks in rural clinics becomes critical in the nursing shortage. Assessing the user needs, e.g., transportation tasks, is needed to adapt technological support measures effectively and achieve the vision of smart hospitals [23]. Despite the importance of analyzing transportation tasks, there is a notable lack of in-depth analysis regarding nursing transportation duties as emphasized in Section 2. Furthermore, the interest in healthcare robotics is growing in the HCI community, even though many existing robots do not fully align with nurses’ needs, and existing research is focused on technical feasibility [18,23].

Therefore, we aim to bridge the gap between the user-centric development of robots, particularly in the unique context of the German nursing system. Our study contributes to the current research gap about clinical nursing transport tasks’ complexities, time allocation, and requirements. We provide the foundation for further research implementing user-focused robots to ease staff from everyday transportation tasks.

## 2. Related Work

Research focuses on overall task assessments and less on nurses’ transportation needs.

### 2.1. Time and Motion Studies

Time and Motion Studies (TMS) aim to optimize industrial processes, with applications emerging in healthcare [24]. According to Klein et al. [25], TMS can uncover optimization and improve efficient work systems. Using the German REFA method, which offers precise work and process measurements, we focused on its application in healthcare tasks [25,26]. Despite its growth, TMS research in healthcare is limited, often due to small sample sizes and self-reported data [27,28]. Westbrook [29] highlighted the relevance of nurses’ patient interaction for health outcomes and acknowledged the extensive time nurses spend on non-direct care tasks. Research shows variability in nursing tasks depending on clinic settings, with a significant portion dedicated to transportation tasks, especially patient transfers [13,30,31]. Lim et al. [31] state that 10.5% of nursing work time is spent on transportation, like patient transfer to diagnostics, food delivery, and drug supply.

However, nurses are also burdened with myriad tasks, such as documentation, care organization, and administration, which do not involve direct patient care [29,32]. The TMS by Michel et al. [33] states that less than one-third of nurses’ work time is spent with patients directly. Hendrich et al. [34] affirm that administrative tasks, particularly documentation, constitute over 27.5% of nursing work time. Conversely, Roche et al. [35] challenge these assumptions, contending that nurses spend most of their time assisting service staff and nursing support workers in direct care tasks [29,35]. Notably, some clinics lack support staff, leaving nurses with continued responsibility for non-direct patient care tasks [36]. TMS may vary based on specific clinic settings [24]. Furthermore, nursing tasks are multifaceted and often involve intra-clinic transfers, such as moving patients for treatments or diagnoses [30]. These transportation tasks warrant a deeper look, given that Fiedler et al. report that over 7% of nursing time is dedicated to patient movement and transfer activities [13]. Fiedler et al. do not account for transport tasks, suggesting that the proportion of daily nursing work spent on transport is higher.

### 2.2. Transportation Needs

Studies by Fiedler et al. [13] and Blay et al. [15], focus on clinical task assessments, with few results about clinical transportation [36]. Nevertheless, transporting medical goods and patients is integral to the efficient execution of clinical nursing practices [17,37,38]. As per the studies by Fiedler et al. [13], Kriner et al. [39], Ryoo et al. [40], and Lim et al. [31], certain categories of goods—namely medical supplies, non-medical equipment, patients, and medical waste—are transported more frequently. Moreover, drug distribution is the most time-consuming good, according to Graf et al. [41] and Ahtiainen et al. [20]. Furthermore, Hendrich et al. [34] identified measuring device transportation and waste handling as time-consuming tasks. Rosenberg et al. [30], Blay et al. [15], and Roche et al. [35] underscore the importance of patient transfers, characterizing them as necessary to avoid decubitus ulcers and respect patients’ mobility needs. Communication is often needed during patient transfer, which implies that transfers are time-intensive [35]. Nevertheless, transporting patients can happen to a nurse 40 times a shift each, which can also be demanding for patients who are not overweight, according to Mukai et al. [14]. Ryoo et al. [40] pointed out that manual patient transfers occur frequently and may be a health risk.

As Table 1 shows, clinical transportation needs are multifaceted, marked by diverse requirements that complicate researching these topics [40]. Existing studies, non-comparable and minor populations, present varying results [29,30]. Furthermore, the time required for transport between studies varies [31]. Fiedler et al. [13] affirm the non-repetitive nature of nursing tasks, underscoring daily variability due to varying patient needs. Concurrent schemes, such as patient communication during transportation or interruptions due to unexpected events, further complicate these tasks [42]. Therefore, more research is warranted to alleviate transportation tasks’ burden and devise effective technical solutions [43].

### 2.3. Assistive Robots for Healthcare Transports

Recent advancements in robotics, especially computational capabilities, have broadened the potential for applying robotic technology in healthcare [18]. Despite these developments, the healthcare sector has been slow to adopt robotic solutions widely [19]. The terminology in this field is varied, with healthcare transport robots being categorized as logistic, service, serving, carrying, and hospitality robots, all falling under the broader classification of assistive robots [18,22,43,44,45,46,47]. However, there is a notable gap in the availability of carrier robots specifically designed for hospital wards [14]. Large logistic robots, typically used for tasks like bed transportation, are limited in hospital environments due to their size and difficulty navigating confined spaces [18,47]. These robots, primarily industry-derived, are not tailored for patient interaction and struggle in narrow hospital corridors and rooms. More miniature robots from the gastronomy sector are being repurposed for healthcare, with models like Keenon’s T5, TUG, and Plato adapted for food transport, capable of functioning in the constrained spaces of hospitals [18,22,43].

Key features for healthcare robots include autonomous navigation, obstacle avoidance, secure compartments, and an integrated tablet for operation [46]. Ohneberg et al. [19] emphasize that current healthcare robots are in developmental and experimental stages. There is a need for research and data focused on nurses’ transportation tasks to develop robotic solutions better suited to the unique environment and demands of hospital wards.

## 3. Methodology

Our study adopts a cross-sectional participatory observational design with two distinct data points, focusing on the transportation needs and execution processes of clinical nurses in rural clinics. This approach provides valuable contributions to addressing the research gap in this area. The first data point was collected through participatory observation conducted from 3 to 9 July 2023 in one rural clinic. A second, separate data point was gathered similarly in another rural clinic from 4 to 10 September 2023. This two-point cross-sectional method enables a comparative analysis of the practices and challenges in different rural healthcare settings

### 3.1. Objectives and Research Questions

We aimed to analyze nurses’ transportation tasks to enable a user-centric development of further assistive transport robots. We aimed to identify the most time-consuming, costly, and common transportation tasks. Furthermore, we identify the allocation and kinds of nursing transports. Our observational study answered the central question: “which transportation needs are undertaken by nurses in clinical settings, and what are their time allocations, requirements, and economic implications?”.

This includes the following subordinate research questions (RQs):

RQ1: Which items are transported, how often, and in which direction?
RQ2: How long does it take to move items, and which requirements exist?
RQ3: Which specific requirements for certain transportation need to be fulfilled?
RQ4: Do transportation tasks vary between different days, shifts, and locations?
RQ5: What is the economic perspective on clinical transportation tasks?

### 3.2. Inclusion and Exclusion Criteria

Our study focused on formal, professional nurses with completed training, working in non-intensive care wards of two rural hospitals in Northern Bavaria, Germany. The analysis included nurses actively working in geriatric and surgical units, providing a diverse perspective on transportation tasks. We included day-shift nurses and excluded night-shift nurses because fewer transports occur at nighttime, as in the pre-test.

Moreover, nurses in training or apprenticeship were not observed, ensuring a focus on experienced nursing practices. Furthermore, we excluded nursing support staff, medical staff (physicians, physician assistants), and other hospital employees involved in ancillary services, like kitchen, cleaning, and pharmacy, from our observation. Focusing on hospitals, we exclude other healthcare facilities, like long-term elderly care.

### 3.3. Study Setting and Sampling

We observed clinical nurses’ transportation tasks in two rural clinics of basic-, regular- and emergency care in Lower Bavaria, Germany. The observations were strategically scheduled: the first observation from 3 to 9 July 2023, in a geriatric nursing unit, and the second from 4 to 10 September 2023, in a surgical unit. These periods were chosen to provide a comprehensive overview of the transportation needs in different nursing contexts. Therefore, our study setting consists of …

1. a geriatric nursing unit for the first observation period (30 mostly immobile patients with an average age of 65 mostly in 17 fully occupied rooms, 30 employees in the nursing staff rotating in a three-shift-system, and additionally one station secretary, three nursing trainees, and two interns),
2. a surgical unit for the second observation (31 patients in 17 rooms capacity, occupied with 12 patients with an average age of 49 years, 28 employees in the nursing staff rotating in a three-shift system, 1 station secretary, 4 nursing trainees).

Both clinics, Arberlandklinik, Viechtach, shortened as VIE, and Kliniken Am Goldenen Steig, Freyung, abbreviated as FRG, are typical rural healthcare facilities in Germany, each employing over 1000 staff members and servicing around 40,000 patients annually. These facilities were integrated into our project “Smart Forest-5G Clinics” (https://www.smartforest-5g.de, accessed on 10 January 2024), chosen for their representativeness of the typical rural healthcare environments across Bavaria.

The study population comprised fully trained day-shift nurses from both hospital wards. The average age of the staff in both clinics was 42 years. The day-time shifts in these clinics were divided into an early shift from 6:00 to 13:30 and a late shift from 13:30 to 21:00. The early and late shifts consisted of 4 nurses. One nurse was randomly selected and observed each shift. Nurses on duty during these shifts were chosen for observation through a random drawing process to ensure a diverse and unbiased representation of nursing practices and transportation dynamics within these rural healthcare settings.

### 3.4. Study Instrument (Android Observation Application)

Our data collection form was based on established literature. For measuring the transportation methods, we utilized the REFA-method [26] and time and motion studies [29,31,33,34,35]. Furthermore, we integrated findings from Fiedler et al. [13] and Blay et al. [15] about transportation in clinical nursing, visualized in Table 1, to derive our observation categories. These clusters and associated categories were inspired by similar studies [13,15] in the field, ensuring relevance and comprehensiveness. Table 2 also visualizes the clusters of transported goods in the last. For instance, ‘Non-Medical Supplies’ included tableware, hair dryers, and cushions, ‘Medical Supplies’ included wound dressings, diapers, and needles, and ‘Pharmacotherapy’ all items regarding medication, infusion, and transfusion.

Three preliminary pre-tests involving a total of six nurses were conducted to test the reliability of our survey. Feedback from these nurses led to revisions in the survey’s clarity, wording, and overall structure. Furthermore, these pilot tests allowed us to critically evaluate the instrument’s practical application, leading to a necessary reduction in variables for feasibility and effectiveness. For instance, we consolidated sub-items, such as transportation goods, into functional clusters that were confirmed during pre-tests.

Despite our modifications and limitations, described in Section 5, it was confirmed that the survey instrument can effectively address the research questions. Our final study instrument included nominal and metrical variables, which were extended by the possibility of adding open-ended data. We used an Android app with a standardized protocol consisting of the variables shown in Table 2. Our application had pre-set categories and allowed free text (open-ended data) for consistent data collection.

### 3.5. Data Collection and Annotation Process

We conducted a participatory observation over seven days in each rural clinic during typical weeks in July and September 2023, excluding public holidays. Observations covered the early (06:00–13:30) and late (13:30–21:00) shifts from Monday to Sunday. Each day, we focused on observing two randomly selected daytime nurses per clinic, using an Android app equipped with a standardized protocol, with the UI visualized in Appendix B. Our approach allowed for precisely tracking nurses’ transportation start and end times.

A crucial aspect of our data collection was maintaining uniformity in observations and ensuring consistent conditions across all settings. Observers were trained and provided with a guide and glossary to support data collection. The glossary also contained information about the transported goods and their clusters, as shown in Table 2. The app also included tooltips to clarify items for observers. All observations were anonymized through the random assignment of nurses. Observers followed the nurses at a safe distance, respecting the privacy of all individuals involved, and refrained from recording any personal data. They were instructed not to interact with nurses, visitors, or patients during observation periods to avoid distraction and bias during transportation. Furthermore, observers avoided entering patient rooms, as necessary data were obtained by noting items like incontinence materials and the time nurses left the rooms. As we used informed consent, information posters were displayed in the clinics to inform about the study, and a declaration of consent was given to all nurses.

### 3.6. Data Analysis

We utilized quantitative data analysis and a costs-benefit calculation based on the literature. First, our data were prepared for evaluation, including deleting incomplete values, performing plausibility checks, and transforming, i.e., summarizing necessary variables. To organize the data, we used Python Pandas. The evaluation of times was based on the REFA-Methodology [48], with some adaptations to our needs according to Table 3.

Our measurements began when a nurse took an item in their hand and ended once the item was handed over and the transport was completed. We evaluated the frequencies of all variables and utilized charts, histograms, scatter, and box plots to represent our results. In addition to the descriptive data analysis, we conducted a template analysis of all free-text comments. The free text was filtered and meticulously coded to ensure the integrity and relevance of our findings. After assessing the coded information, it became evident that the comments provided a summarizing overview and a deeper understanding of the data, complementing our quantitative analyses. Furthermore, with the factual transport time, visualized by Table 3, we calculated the transport costs by considering the average hourly nursing wage and projected it for a whole year. The calculation is detailed under Section 4.8.

## 4. Results

This section presents the results related to the transportation tasks within the two rural clinics in Germany. These outcomes were derived from a comprehensive participatory observation conducted over two distinct periods within 2023, aiming to shed light on the transportation tasks of clinical nurses during day shifts. Initially, we summarize the transportation needs, the duration, the medium, and the requirements for both clinics.

### 4.1. Overview of Transported Goods

During each daytime shift, one nurse was observed. A total of 1830 transports were recorded in both clinics for two weeks. The transport distribution is shown in Table 4:

It becomes apparent that clinical nursing involves transporting a diverse range of goods. We have grouped these into 10 categories for clarity and ease of interpretation. As illustrated in Table 4, Non-Medical Supplies emerged as the predominant category, making up nearly a third (27.05%) of all transported items. Medical Supplies closely trailed this, such as wound dressings, which represented 17.32% of the overall count. Pharmacotherapy formed another significant portion, amounting to 14.10%. Further, more specialized categories like medical devices accounted for 12.51%, and meals or drinks for 12.68%. Venturing into the less frequent items, which each represented less than 5% of the total, we observed documents (4.10%), patient transfer aids (3.99%), miscellaneous items (4.81%), patients (2.89%), and lab samples (0.55%). It is worth noting that the distribution of transported goods is inconsistent across rural clinics, Freyung (FRG) and Viechtach (VIE). There are more transportation tasks in the clinic of VIE, especially since there is more transportation in the category of non-medical supplies, with 338 cases, compared to FRG, with 157 cases. Furthermore, more meals and drinks are transported in FRG as in VIE.

### 4.2. Frequency of Locations

Table 5 provides the transportation start and endpoints. Six transports were canceled, explaining why the total numbers of start and end locations differ. The primary focus of transport is the corridor, patient rooms, and the station office. With 36.69%, the corridor is the most common starting location. A total of 27.95% of transports started further from the patient room and 25.75% from the station office. Other areas, having percentages under 5%, highlight that transport between places like the kitchen or the storage is less common. When observing the termination points, the patient room dominates with 50.30% and is followed by the corridor (21.70%). Furthermore, the station office again emerges as an important location, making up 8.91% of the end locations. Areas such as the Lounge and various miscellaneous locations, encompassing kiosks, diagnostics areas, and elevators, although constituting smaller percentages, point to specific and specialized transportation needs. In summary, transports focus on the corridor, patient rooms, and the station office.

### 4.3. Transport Duration Analysis

Another angle of understanding the efficiency and potential bottlenecks in transportation tasks is by assessing the duration taken for transports.

#### 4.3.1. Cumulative Distribution of Total Transport Duration

**Figure 1:**
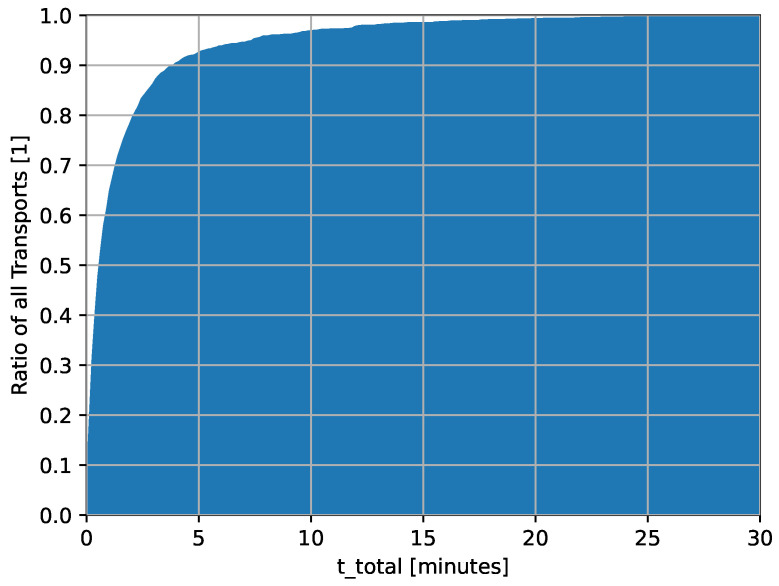
Cumulative total transport duration, histogram in minutes.

In Figure 1, the cumulative distribution of total transport duration ($t_{\mathrm{total}}$), including all interruptions and required setup time, is illustrated through a histogram for all transported items. More than 90% of all transports were completed in under five minutes.

The mean transport duration is 1.82 min, with a standard deviation of 3.47 min. In total, 1% of transports finish in 4 s, while 10% are complete within 11 s and 25% within 20 s. The median is 0.70 min, signifying that half of the transports are concluded under that duration. Notably, even including the interruptions and setups, 90% of all transports are finished in less than 4.05 min. While the majority of transports demonstrate efficiency, some extremes stand out. Specifically, the longest 1% of transports (p99) reach up to 17.72 min. The maximum transport duration has extended up to 44.58 min. These outliers above the 99th percentile highlight the complexity of clinical nursing.

#### 4.3.2. Setup Times

**Figure 2:**
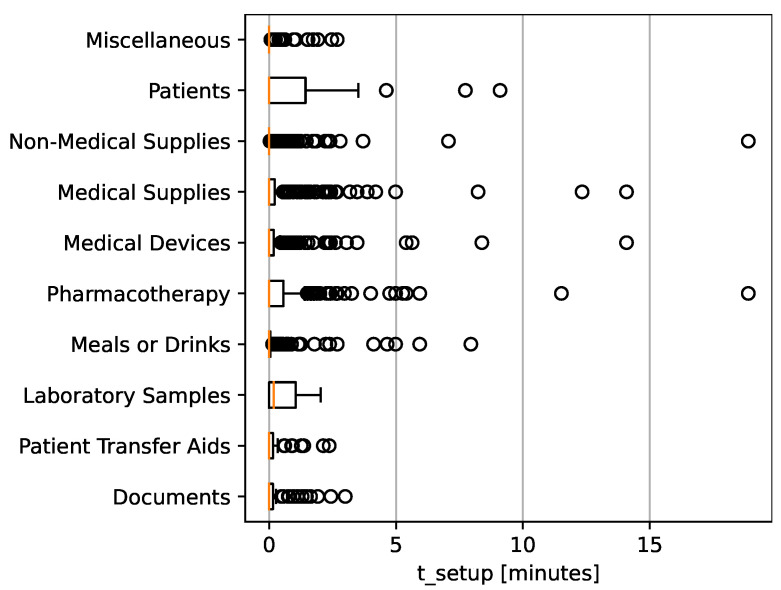
Transported goods and setup time, boxplots with outliers in minutes.

The setup time ($t_{\mathrm{setup}}$), referring to preparatory steps like disinfecting or turning on a system before and after initiating a transport, averages 0.31 min. While 25% of the transports (Q1) did not require any setup time, 75% of the transports (Q3) necessitated up to approximately 7 min. Many transports require minimal to no setup. The longest observed $t_{\mathrm{setup}}$ was 18.88 min. As depicted in Figure 2, patient transports, pharmacotherapy, and lab sample transports often necessitate preparations.

#### 4.3.3. Interruptions during Transports

Analyzing interruptions ($t_{\mathrm{inter}}$) during transportation, we find an average $t_{\mathrm{inter}}$ of 0.74 min with a standard deviation of 2.49 min. Most transports are efficient: up to the median, meaning no interruptions exist. By Q3 (75%), $t_{\mathrm{inter}}$ rises to 0.25 min. However, extreme cases exist, with the most extended interruption being 35.17 min. Such delays beyond the 99^th^ percentile highlight challenges in clinical nursing’s transportation, such as emergencies or equipment issues.

#### 4.3.4. Transported Goods and Their Factual Transport Time

**Figure 3:**
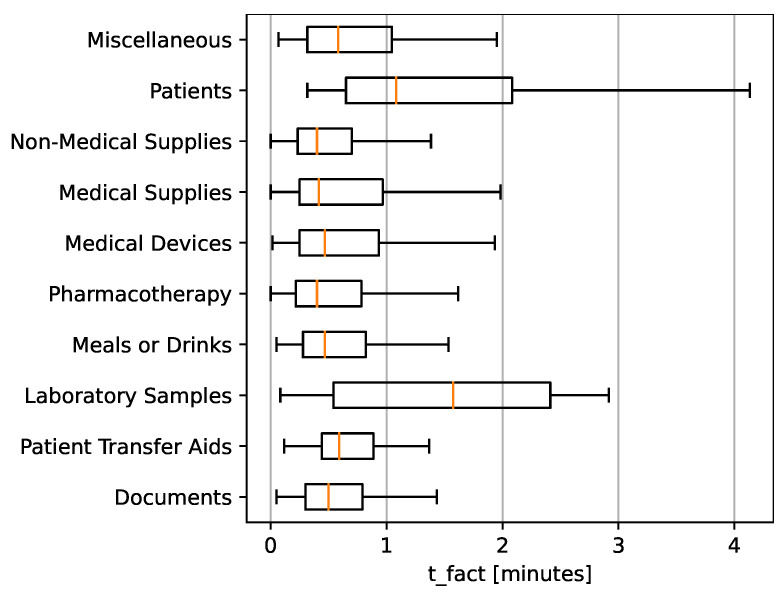
Transported goods and factual transport time, boxplots without outliers in minutes.

The factual transport time ($t_{\mathrm{fact}}$), the time without any interruptions and set-up, is important for understanding transportation efficiency. Figure 3 showcases a box plot.

Patient transport and lab samples stand out among the items shown in the boxplot. Not only do they have a higher median factual transport time compared to other items, but they also exhibit a broader range in their distribution. This suggests that these two categories face more variability in transportation time. The extended duration for patient transport could be attributed to the inherent complexity and care required in moving patients. Similarly, transporting lab samples might involve specific protocols or detours, leading to longer transportation times. The median factual transport time ($t_{\mathrm{fact}}$) for most transported goods, including non-medical supplies, medical supplies, medical devices, pharmaceuticals, meals or drinks, patient transfer aids, and documents, falls under a minute. Moreover, even their interquartile ranges (Q1 to Q3) suggest a tight distribution, mainly concentrated within this under one-minute duration.

Contrastingly, patient transfers and lab samples stand out in the box plot. While predominantly registering under a one-and-a-half minute for their median times, these two categories have a notably wider spread in their box plot. Furthermore, the whiskers are wider, especially in patient transport, indicating greater variability in $t_{\mathrm{fact}}$. Specifically, up to the Q3, patient and lab transports exhibit times ranging between two and three minutes.

### 4.4. Transport Durations over a Week

**Figure 4:**
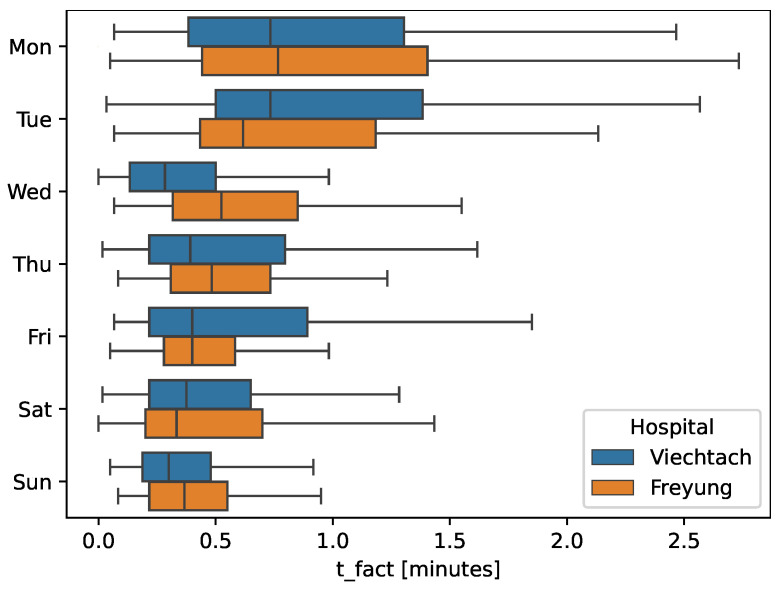
Factual transport duration and weekdays, boxplot grouped by clinics.

As demonstrated in Figure 4, there’s consistency in the distribution of transports throughout the week. There is not any particular day where apparent anomalies occur regarding transport duration or frequency. Whether on weekdays or weekends, the transportation workflow maintains a steady pace throughout the observation period. At the beginning of the week, the duration and outliers in $t_{\mathrm{fact}}$ are more comprehensive.

**Figure 5:**
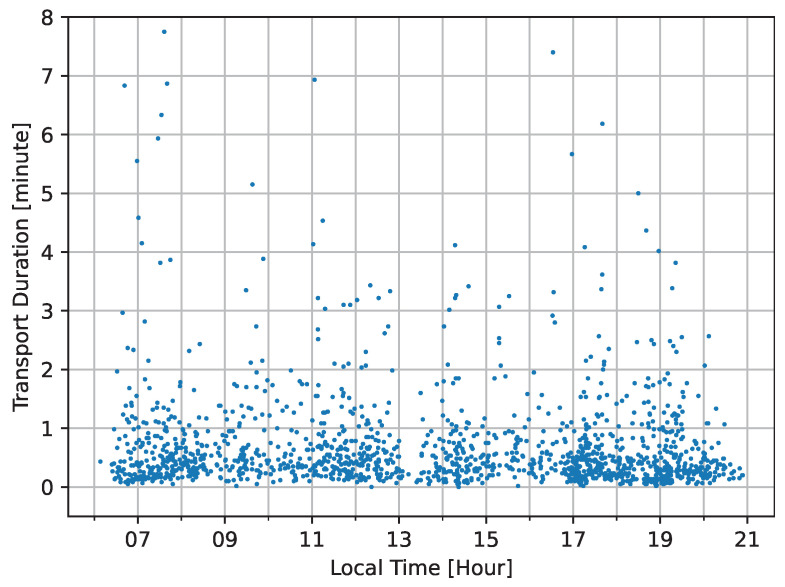
Scatter plot of factual transport duration and local time.

Delving into daily trends, Figure 5 shows the spread of transports throughout the day. The data do not present any sharp spikes at specific hours. However, a mild surge in transport can be observed during the early morning hours between seven and eight o’clock and again in the late afternoon, from 6.30 to 8 a.m. These could be times when routine medical activities or shift changes necessitate more movement. Furthermore, another slight surge is in the late afternoon, from 5 to 7 p.m., which can be related to dinner. Apart from these periods, the transports are evenly distributed.

### 4.5. Share of Transport Mediums

Our analysis shows variations in the transport mediums across the clinics (Table 6). The most prevalent medium was ’By Hand,’ accounting for 77.15% of all transports. Other transport mediums followed, including the Nursing Trolley (13.06%) and Tray (11.73%). Wheelchairs, beds, cardboard, and others constituted less than 7% combined.

As most items were transported by hand (77.15%), a detailed examination of this category sheds light on the nature of items and reasons for this preference. The most frequently hand-carried items were Non-Medical Supplies, with 328 cases (28.32%), and Medical Supplies at 263 cases (22.71%). Pharmacotherapy items were also prominently hand-carried, totaling 226 cases (19.52%). Meals or drinks and medical devices made up 160 (13.82%) and 156 (13.47%) cases, respectively. Less frequent hand-carried items included documents with 70 cases (6.05%), Miscellaneous items at 62 (5.35%), and patient transfer aids at 35 (3.02%). In 17 instances (1.47%), patients were “hand-carried” in the sense of being taken by the hand and led to their destinations. Lab samples were the least transported by hand, tallying at seven cases (0.60%).

### 4.6. Transportation Requirements

In 1291 (79.25%) of all transports, no specific requirements were noted. Collection was necessary in 4.73% of cases, while 4.30% needed nursing supervision during transport. Disinfection and observation of medication intake were required in 2.89% and 2.82% of transports, respectively. In 2.15%, patients needed general help taking drugs, food, or mobilization during transports. Bio-hazard precautions or isolation were needed in 1.41%. Less than 1% of the transports had other requirements, such as safety, weight considerations, urgency, labeling, elevator use, and temperature control.

### 4.7. Differences between the Clinics: Geriatric (VIE) vs. Surgery Unit (FRG)

Table 7 contrasts the top transported goods in each clinic. Viechtach (VIE) features a geriatric unit, whereas Freyung (FRG) operates a surgery unit, impacting transportation and patient mobility. Notably, FRG’s surgical unit sees more patient transports due to more frequent examinations. FRG’s geriatric unit transported fewer non-medical supplies (19.50%) compared to VIE’s 32.98%. Conversely, FRG had more meal or drink transports (20.87%) versus VIE’s 6.24%. Patient transports were more frequent in VIE (3.90%) than in FRG (1.61%). Moreover, lab transports were exclusive to VIE.

In addition, FRG has more prolonged interruptions than VIE, but VIE has extended setup times. Average transport duration is closely matched, but VIE displays a broader duration range. At the median for $t_{\mathrm{fact}}$, FRG’s transports are 0.47 min, slightly longer than VIE’s 0.42 min. Detailed time breakdowns are available in the Appendix A and Figure 4.

### 4.8. Transportation Cost Analysis

Using the direct transport time, $t_{\mathrm{fact}}$, which omits setup and interruption times, we analyzed the costs over a two-week observation period in two clinics, focusing on two nurses per daytime shift. As depicted in Table 8, these observations revealed a total transportation cost of 514 €, corresponding to 24.73 h of work. It’s worth noting that for the calculation, the hourly rate of 20.79 € was derived from the German collective agreement TVÖD-P [49], which sets a nurse’s gross wage at 3532 €, inclusive of 600 € for additional employer costs, such as social insurance. Our data, comprehensive for the day and late shifts with typically four nurses each, does not cover the night shift.

A portion of transport costs is attributed to the transport of ’meals or drinks’, taking up 4.81 h and accounting for approximately 99.96 € during the observation period. Given the clinic’s staffing structure, with an average of four nurses operational during each daytime shift, this translates to a monthly transport cost of 799.68 € solely for meals or drinks on the observed stations. When projected over a year, this single task accumulates an annual expense of around 9596.16 € across both clinics.

## 5. Discussion

Our study fills a critical gap in understanding transports in nursing [27,36], focusing on clinical nurses and analyzing their time use, requirements, and economic impacts.

### 5.1. Methodical Limitations

Our participatory observation method, while robust, is subject to certain limitations and potential biases. Our focus was primarily on clinical nurses, driven by higher wages and staffing deficits. Consequently, this narrowed lens did not encompass the activities of service support staff, who may play a significant role in transport tasks. Additionally, the inherent risk of observational studies, where participants may alter their behavior when they know they are being observed, remains a concern. As cited in [50], the Hawthorne effect underscores this issue, and multiple observers’ involvement could potentially amplify it. We employed random participant selection to mitigate these biases and utilized a standardized, app-driven data collection method.

Furthermore, our method incorporated the REFA-Methodology [48], initially designed for production and industry. While it sharpened our time measurements, its limitations might not perfectly align with modern healthcare dynamics, given its manufacturing roots and age. The study instrument is oriented around existing nursing transportation studies, like Fiedler et al. [13] and Blay et al. [15]. We adapted and needed to shorten the variables, so we clustered transported goods into groups. Therefore, our methodology did not allow for an in-depth examination of the specific types of items transported, which could have provided more granular insights into clinical transportation needs. While this would have been valuable, it was not practicable within the scope of our study due to logistical constraints and the need to maintain a feasible and manageable data collection process.

Furthermore, the validity and generalizability of our findings are limited by our study’s specific settings and short duration [51]. Unique characteristics of our observation sites, such as FRG’s lower patient occupancy and the presence of interns, influenced our results. External factors, including the particularities of student internships and clinic-specific processes, may hinder the generalizability of our findings, indicating a need for broader research across diverse settings.

Despite these limitations, our study provides cost-effective and pragmatic insights into rural clinical transportation challenges, suggesting potential avenues for innovation, such as using transport robots and paving the way for further research.

### 5.2. Transportation Tasks and Durations

Our findings corroborate with prior research, identifying various clinical transport tasks, including medical supplies, non-medical equipment, patients, and medical waste [13,39,40]. We have further identified transport categories, such as distributing food and drinks, which are not widely discussed in the existing literature. Transportations like patient transfers, likely due to their inherent complexity, consume more time in our study, aligning with the insights of Rosenberg et al. [30], Blay et al. [15], and Roche et al. [35]. Nevertheless, it is limiting that we clustered primarily the transported goods, with the need for further studies to be more detailed.

Data from Table 4 points out a divergent distribution of transports between our two observed clinics attributed to the unique wards. The dementia ward in VIE, for example, could lead to a high percentage (19.50%) of non-medical goods transports. This suggests a possibility that patients in VIE might have a higher dependency on nursing staff, necessitating more non-medical item transports. Conversely, the lower percentage in FRG could be due to its patients being more independent. These disparities underline the influence of the clinic’s structure and patient demographics on transportation tasks.

During our two-week study, we observed 24.73 h for transports for two daytime nurses. According to Lim et al. [31], approximately 10.5% of nursing work time is dedicated to transportation tasks, such as transferring patients to diagnostics and supplying food and medicines. Our data indicate that nurses working 39 h per week spend 15.85% of their work time on observed transportation tasks, more than Lim et al. [31] reports. Expanding on durations, our study offers a distinct perspective influenced by the REFA methodology, which differentiates between different time components. We highlight the specific durations but abstain from evaluating their efficiency or wasted potential. In summary, our findings resonate with observations on the time-intensive nature of drug distribution [20,41]. Furthermore, Hendrich et al. [34] identified medical device transports and waste management as time-consuming, which we cannot confirm in detail for waste management because of missing subcategorization. Moreover, our observed time intensities for patient transfers align with reports of their inherent complexity from Rosenberg et al. [30], Blay et al. [15], and Roche et al. [35]. Additionally, we identified other time-consuming transports, like food and drinks, which have not been discussed prior.

Figure 5 exhibits rhythmic patterns in transports, possibly tied to daily routines, like the checks and meal times. We found a slight surge in transport between 6:30 and 8 a.m. and 5 to 7 p.m., but apart from these peak hours, the consistent spread of transport events suggests a balanced demand for transport services throughout the day and the week. This might reflect an efficient scheduling and resource allocation mechanism within the clinics.

### 5.3. Mediums and Requirements

Table 6 highlights a reliance on manual transport linked to potential physical strain [40]. Our data lacks specifics like item weight, which influences transport time [40]. Moreover, similar to Westbrook et al. and Peter et al.’s findings, under 5% of all transport tasks involve direct patient interactions, additional care, or collection of items [29,36]. In summary, using robots for transport might also reduce physical strain for nurses and has hardly any special requirements in most cases.

### 5.4. Economical Implications and Assistive Robot Potential

Clinic transports impose economic consequences, corroborated by our study and Fiedler et al. [13]. Our financial analysis is based on actual transport duration, accommodating factors such as disinfection and navigational challenges pertinent to robots. Table 9 shows potential amortization, focusing on automating meal and beverage transports.

Utilizing cost-effective robots like Temi, priced at 5000 €, the investment can be recouped within a year. Projecting further, we calculated the costs for 5 years, presuming the robots’ operational lifespan adheres to German depreciation guidelines. Extending this projection over 5 years suggests more economic benefits. Still, the scientific community and manufacturers know just little about the lifespan of assistive robots in hospital settings, which necessitate the use of disinfection liquids that can probably harm robots, and the distances that robots need to travel can be high.

While our calculations provide a directional guideline, we hinge on the assumption of achieving full automation for meal and beverage transports. Our presumption might be overly simplified. Broadening the scope of robot tasks to encompass additional items or span various wards could enhance operational efficiencies but also necessitates comprehensive research. Considering different robots’ unique capabilities, limitations, and applications in clinical contexts leads to further research needs [18,43].

## 6. Conclusions

### 6.1. Implication for the Nursing Practice and Technicians

Our research highlights transportation as a common and time-consuming nursing task involving non-medical and medical items, notably meals and drinks. Key areas such as corridors, patient rooms, and station offices emerged as major transport nodes, suggesting these as optimal locations for robotic support. A notable 77.15% of transport tasks are manually executed, often involving short, repetitive trips, thereby contributing to the physical burden on nurses. The factual transport time for most items was under a minute, with patient transport and lab samples showing more variability.

We advocate for the widespread adoption of assistive robots to handle routine transportation tasks in healthcare settings, easing nurses’ workloads. It is necessary to monitor market trends to evaluate the impact of more accessible, cost-effective robotic solutions. Recent studies visualized in Section 2 and discussions with robot manufacturers showed that the industry focuses more on our identified primary focus on food transportation [43]. Our research confirms that food transport represents a significant cost factor and that the industry is on the right track with its developments. Some hospitals in metropolitan areas already employ robots for food delivery [19]. Nevertheless, technicians should work on cost-effectiveness for widespread use in financially tight rural clinics. Furthermore, available products are either prototypes or are not specially designed to meet the complex needs of hospital wards, like disinfection and hygiene.

### 6.2. Further Research Directions

The rapidly evolving robotics industry, with new transport robots, should follow evidence-based product design, which alleviates nurses and is user-centered. For healthcare providers considering robot implementation, understanding the impact of hospital demands, such as disinfection and distance traversing, on repair frequency and maintenance costs is vital for accurate cost-benefit analysis and durability assessments in clinical settings. We need to know more about the product lifecycle and its usage.

Furthermore, research should continue to explore the transported goods in different hospital areas and potential synergies to use a robot in other wards to maximize utilization and obtain shorter amortization periods. We recommend initiating intervention studies to investigate how robots can efficiently transport food while identifying limitations. Different robots should be used and compared in those interventions regarding their benefits and barriers. Furthermore, during applied research by our project “Smart Forest-5G Clinics” and the scientific community, we should discover other outcomes connected with implementing assistive transport robots in nursing. This outcome of robots could include alleviating the nurse’s health strain, effects on nurses’ job satisfaction, and if robotics could positively affect the profession’s image. These are underrated topics that are vital in staff shortages and short retention periods in nursing.

## Figures and Tables

**Table 1:** Transportations in clinical nursing, inspired by Fiedler et al. [13] and Blay et al. [15].

Transportation’s	Description
Patient Moving	Transfer and repositioning ^1^, assisting the patient to walk ^1^, moving wheelchair, lift, walker or bed with ^1^ and without patients
Patient Care Transports	moving measuring machines, medication management ^1^, moving bedside table ^1^, laundry delivery and replacing, waste and disposal management, replacing diapers ^1^
Nursing Care Transports	sanitizing and hygiene, bringing safety gown, bringing medical devices and tools, handling vitals equipment, fluids, drugs, hampers, equipment, products, collecting samples ^1^, wound management products and dressings ^1^
Housekeeping	cleaning, disposing of medication packs, dishes, nutrition ^1^, recycling, filling storage racks, assisting with belongings ^1^
Documentation and Charting	answering the phone, transporting documents to the patients for signage, organizing binders, reviewing on the computer

^1^ Direct care tasks according to Roche et al. [35]. Note: this table represents examples and is incomplete.

**Table 2:** Overview of recorded transportation data (variables in the study instrument).

Variables	Description	Examples and Clusters
Timestamp ^1^	Times at the Beginning and End of Transports	Starting with Nurses Taking Transport Goods and Ending with Handover
Set-Up ^1^	Time for Set-Up	Switching on a Device, Collecting Items, Preparing Lab Tubes, Disinfection
Operational ^1^	Time for Operational Interruption	Waiting for a Key or a Device from a Colleague
Personal ^1^	Personal Interruption	Small Talk, Private Calls, Toilet Breaks
Locations	Start-Point, Intermediate Places and Endpoint of each Transport	Corridor, Patient Room, Station, Storage, Kitchen, Lounge, Unsanitary/Disposal, Bath, Lab, …
Goods ^2^	Materials and Items Transported By Nurses	Non-Medical Supplies, Medical Supplies, Pharmacotherapy, Meals or Drinks, Medical Devices, Documents, Patient Transfer Aids, Patients, Lab Samples, Other
Medium	Medium of Transport	By Hand, Trolley, Tray, Wheelchair, Bed, Cardboard, Other
Requirements	Transport Requirements	Collection of Items, Nursing Supervision, Disinfection, Observation of Medication Intake, General Help, Bio-Hazard Precautions, Safety, Weight, Urgency, Labeling, Elevator Use, Temperature
Comments	Additional Information	Open-Ended Data On Transports

^1^ Times variables, collected inspired by the REFA-Methodology [48]. ^2^ Transported goods derived from studies by Fiedler [13] and Blay et al. [15].

**Table 3:** Time categories based on REFA-methodology [48].

Time Category	Acronym	Description
Total Transport Time	$t_{\mathrm{total}}$	Duration from start of setup to complete delivery
Interrupt Time	$t_{\mathrm{inter}}$	Operational + Personal interrupt time
Factual Transport Time	$t_{\mathrm{fact}}$	Total Transport Time − Interrupt Time − Setup Time
Setup Time	$t_{\mathrm{setup}}$	Time to prepare for a task

**Table 4:** Transported goods during the two-week observation by two daytime nurses (N = 1830).

Category	Distribution		By Clinic			
	n	%	Freyung (FRG)		Viechtach (VIE)	
Non-Medical Supplies ^1^	495	27.05%	157	19.50%	338	32.98%
Medical Supplies ^2^	317	17.32%	154	19.13%	163	15.90%
Pharmacotherapy ^3^	258	14.10%	111	13.79%	147	14.34%
Meals or Drinks ^4^	232	12.68%	168	20.87%	64	6.24%
Medical Devices ^5^	229	12.51%	100	12.42%	129	12.59%
Miscellaneous ^6^	88	4.81%	37	4.60%	51	4.98%
Documents ^7^	75	4.10%	42	5.22%	33	3.22%
Patient Transfer Aids ^8^	73	3.99%	23	2.86%	50	4.88%
Patients ^9^	53	2.89%	13	1.61%	40	3.90%
Laboratory Samples ^10^	10	0.55%	0	0.00%	10	0.98%
Total	1830	100%	805	100%	1025	100%

^1^ E.g., tableware, hair dryers, cushions, phones. ^2^ E.g., wound dressings, diapers, needles. ^3^ E.g., medication, infusion, transfusion. ^4^ E.g., breakfast, water, coffee. ^5^ E.g., blood pressure monitors, thermometer, blood gas analysis, scale, suction, defibrillator. ^6^ E.g., items not fitting in categories. ^7^ E.g., doctor’s letters, forms. ^8^ E.g., wheelchairs, beds, lifting slings. ^9^ E.g., transport of patients for procedures. ^10^ E.g., urine, blood, skin swabs.

**Table 5:** Overview of start and end locations of transportation.

	Start Location (n = 1499)		End Location (n = 1493)		
Location	n	%	Location	n	%
Corridor	550	36.69%	Patient Room	751	50.30%
Patient Room	419	27.95%	Corridor	324	21.70%
Own Station (Office)	386	25.75%	Own Station (Office)	133	8.91%
Storage	52	3.47%	Unsanitary/ Disposal	92	6.16%
Station Kitchen	33	2.20%	Storage	69	4.62%
Lounge	21	1.40%	Station Kitchen	42	2.81%
Unsanitary/Disposal	9	0.60%	Lounge	33	2.21%
Other Station	8	0.53%	Other Station	10	0.67%
Nursing Bath	5	0.33%	Laboratory	8	0.54%
Laboratory	4	0.27%	Nursing Bath	4	0.27%
Misc. (Kiosk, Elevator)	12	0.80%	Misc. (Kiosk, Elevator)	27	1.81%

**Table 6:** Overview of the transport mediums used (N = 1629).

Medium	n	%	Freyung	Viechtach
By Hand	1158	77.15%	503	655
Trolley (Nursing Trolley/Disposal)	196	13.06%	120	76
Tray	176	11.73%	85	91
Wheelchair	36	2.40%	8	28
Bed	30	2.00%	15	15
Cardboard	15	1.20%	14	1
Other	18	1.00%	10	8
Total	1629	100%	755	874

**Table 7:** Top 5 transported goods by clinic (N = 1830).

Rank	Freyung (n = 805)		Viechtach (n = 1025)	
	Category	%	Category	%
1	Meals or Drinks	20.87%	Non-Medical Supplies	32.98%
2	Non-Medical Supplies	19.50%	Medical Supplies	15.90%
3	Medical Supplies	19.13%	Medication, In-/Transfusions	14.34%
4	Medication, In-/Transfusions	13.79%	Medical Devices	12.59%
5	Medical Devices	12.42%	Meals or Drinks	6.24%

**Table 8:** Calculation of the costs per transported items for both clinics (two wards, in €).

	Observation Period (2-Weeks)		Costs in € for Early- and Late Shifts ^1^	
Transportations	Sum of Hours ($t_{\mathrm{fact}}$)	Costs	Costs per Month ^2^	Costs per Year ^2^
Meals or Drinks	4.81	99.96 €	799.68 €	9596.16 €
Medical Supplies	4.40	91.48 €	731.84 €	8782.08 €
Non-Medical Supplies	4.33	89.92 €	719.36 €	8632.32 €
Medical Devices	3.29	68.40 €	547.20 €	6566.40 €
Pharmacotherapy	3.16	65.69 €	525.52 €	6306.24 €
Patients	1.41	29.31 €	234.48 €	2813.76 €
Miscellaneous	1.29	26.82 €	214.56 €	2574.72 €
Documents	1.16	24.12 €	192.96 €	2315.52 €
Patient Transfer Aids	0.63	13.10 €	104.80 €	1257.60 €
Laboratory Samples	0.25	5.20 €	41.60 €	499.20 €
Total	24.73	514.00 €	4112.00 €	49,344.00 €

^1^ We used an hourly nursing wage of 20.79 € for calculation, orienting on the TVÖD-P [49]. ^2^ Projections

**Table 9:** Economic evaluation of robots in transporting all meals and drinks in the clinics.

Model	Cost for 2 Robots (€)	Amortization (Years)	Savings after Amortization (€) ^2^
Keenon T5 ^1^	30,000 €	3.13	17,945 €
Keenon W3 ^1^	40,000 €	4.17	7965 €
Temi ^1^	10,000 €	1.04	38,001 €
Plato ^1^	18,000 €	1.88	29,940 €

Assumption of transporting all meals and beverages in two wards. ^1^ Prices according to www.robotemi.com, www.keenonrobot.com and www.robshare.de, accessed on 12 January 2024. ^2^ Five-year usage, without service

**Figure A1:**
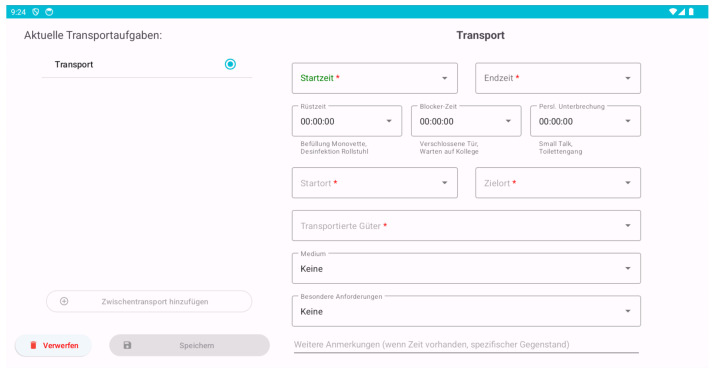
Transported goods and factual distribution time, boxplots without outliers.

## Data Availability

Our research data are available from the authors and will be provided on request due to privacy/ethical restrictions. Furthermore, additional information about our project is available via www.smartforest-5g.de (accessed on 24 January 2024).

## Public Involvement Statement

No public involvement in any aspect of this research.

## Guidelines and Standards Statement

This manuscript was drafted against the Guidelines for Safeguarding Good Research and Practice, available via www.wissenschaftliche-integritaet.de/en/code-of-conduct (accessed on 24 January 2024).

## Abbreviations

The following abbreviations are used in this manuscript:

UC	Use-Case
VIE	Hospital Viechtach
TMS	Time and Motion Studies
Q1	First Quartile
FRG	Hospital Freyung
HCI	Human-Computer Interaction
Q3	Third Quartile
RQ	Research Questions

## Appendix A. Different Times Oriented on REFA in the Two Clinics

The clinic FRG records a longer total transport time than VIE. When examining the times in detail, there are differences. In FRG, more prolonged interruptions are observed compared to VIE, which, on the contrary, has more extended setup times. While both clinics demonstrate almost identical mean factual transport durations ($t_{\mathrm{fact}}$), with FRG at 0.76 min and VIE at 0.77 min, the variability of these durations, as depicted by the standard deviation, is notably higher in VIE (1.76) than in FRG (1.05). This suggests that while VIE experiences a broader range of transport durations around its mean, FRG’s durations are more clustered around its average. Furthermore, the quartile analysis provides a clearer picture of the $t_{\mathrm{fact}}$. A total of 50% of the transports in FRG have a $t_{\mathrm{fact}}$ less than 0.47 min, while in VIE, the median is marginally lower at 0.42 min. Moreover, in Q3, the transport durations are closely matched, with 0.82 min in FRG and 0.80 min in VIE.

**Table A1:** Transport duration comparison between freyung and viechtach clinics.

	Clinic	Mean	Std	Q1 (25%)	Q2 (50%)	Q3 (75%)
$t_{\mathrm{total}}$	Freyung	2.24	4.01	0.43	0.90	2.28
	Viechtach	1.49	2.94	0.28	0.58	1.48
$t_{\mathrm{inter}}$	Freyung	1.32	3.34	0.00	0.00	1.08
	Viechtach	0.29	1.35	0.00	0.00	0.00
$t_{\mathrm{setup}}$	Freyung	0.16	0.56	0.00	0.00	0.00
	Viechtach	0.43	1.38	0.00	0.00	0.28
$t_{\mathrm{fact}}$	Freyung	0.76	1.05	0.28	0.47	0.82
	Viechtach	0.77	1.76	0.22	0.42	0.80
